# Chromosome‐Level Reference Genome of an Endemic, Endangered Long‐Armed Scarab (*Cheirotonus formosanus*): Discovery of a Putative Y‐Linked Scaffold and Demographic History

**DOI:** 10.1002/ece3.73483

**Published:** 2026-04-14

**Authors:** Sean Chien, Jen‐Pan Huang, Heath Blackmon

**Affiliations:** ^1^ Department of Biology Texas A&M University College Station Texas USA; ^2^ Biodiversity Research Center Academia Sinica Taipei Taiwan; ^3^ Ecology and Evolutionary Biology IDP Texas A&M University College Station Texas USA

**Keywords:** coleoptera, endangered species, endemic species, genome assembly, Y chromosome

## Abstract

We present a high‐quality, chromosome‐level reference genome for the endangered, endemic long‐armed scarab beetle *Cheirotonus formosanus* from Taiwan. Using PacBio HiFi and Hi‐C data, the nuclear assembly spans 600.9 Mb with *N*_50_ of 69.5 Mb (largest scaffold 93.2 Mb). BUSCO completeness is 99.3% for the assembly and 97.7% for the genome annotation. The circularized mitochondrial genome (20,286 bp: GC content 30.74%) contains the canonical 37 genes, and HiFi long reads resolve tandem repeats in the control region that are intractable with short read platforms. A chromosome‐quotient approach with male (PacBio HiFi) and female (Illumina) reads assigned nine autosomal scaffolds, one X‐linked, and a putative Y‐linked scaffold (1.1 Mb). Historical demographic inferred independently from male and female genomes reveal identical trajectories. These results show a moderate effective population size (*N*_*e*_) through most of the last ~500 thousand years ago (kya) and increase beginning ~115 kya with a peak around ~50 kya, followed by a decline toward the Last Glacial Period and relative stability in the Holocene. Recent demographic inference and analysis of runs of homozygosity (ROH) indicate that while *N*_*e*_ has remained low, the genomic landscape is dominated by short homozygous segments (< 1 Mbp). The absence of very long ROH (> 5 Mbp) suggests that current inbreeding levels result from long‐term historical restriction rather than recent consanguineous mating. Together, these resources and workflows enable sex‐chromosome characterization, comparative mitochondrial genomics, and cross‐timescale demographic inference in *C. formosanus* and provide reusable pipelines for beetle genomics and conservation.

## Graphical abstract

Male and female *Cheirotonus formosanus*. Adults have metallic dark‐brown elytra with scattered light‐brown spots and golden hairs covering the ventral thorax and abdomen. Body length ranges from ~50 to 65 mm and males exhibit exaggerated, elongated forelegs.

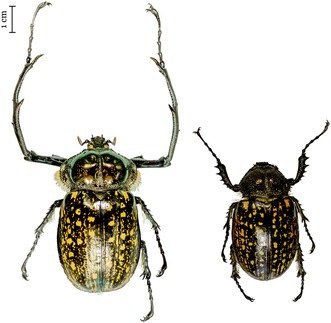

## 1 Introduction

Insects play key roles in ecosystem function through pollination, nutrient cycling, and food web support, yet many taxa show sustained declines in abundance and diversity (Vogel 2017). Habitat loss and fragmentation driven by anthropogenic activities and climate change reduce connectivity among populations and reduce genetic diversity (Wagner 2020). These pressures have negative impacts on effective population size (*N*_*e*_), which increases drift and inbreeding, and diminishes adaptive potential. Those effects are especially strong in small isolated populations such as island and montane endemics. Conservation planning increasingly relies on genomic evidence to quantify diversity, connectivity and demographic history and to prioritize management actions. Genomic data have already facilitated successful interventions. For example, genetic rescue in the Florida panther used genome‐wide markers to guide translocations that increased heterozygosity and reduced inbreeding depression (Johnson et al. 2010). Conversely, the Chinese giant salamander case illustrates how failing to recognize cryptic diversity can undermine conservation (Yan et al. 2018). Despite this promise, most threatened, non‐model organisms still lack high‐quality reference genomes, limiting the resolution and reliability of downstream inference.

Recent advances in long‐read sequencing (e.g., PacBio HiFi, Oxford Nanopore) and Hi‐C scaffolding have made high quality and chromosome‐level assemblies for non‐model organisms computationally tractable and affordable. Such references substantially improve SNP and structural variant discovery tremendously by increasing the contiguity and reducing mapping ambiguity. They also resolve complex repeat regions that confound short read assemblies, including the mitochondrial control region and repeat rich sex‐linked chromosomes. Reference genomes also allow mapping based reuse of fragmented DNA from museum specimens and reduce possible biases from reduced‐representation datasets (e.g., ddRAD seq) by generating comparable markers across samples. Diploid whole genome data support long term demographic inference, with PSMC or MSMC recovering long term *N*_*e*_ trajectories and methods such as GONE2 estimating recent *N*_*e*_ from SNP panels (Li and Durbin 2011; Mather et al. 2020; Santiago et al. 2025). These capabilities are particularly valuable for endangered species with limited genomic resources.

**FIGURE 1:**
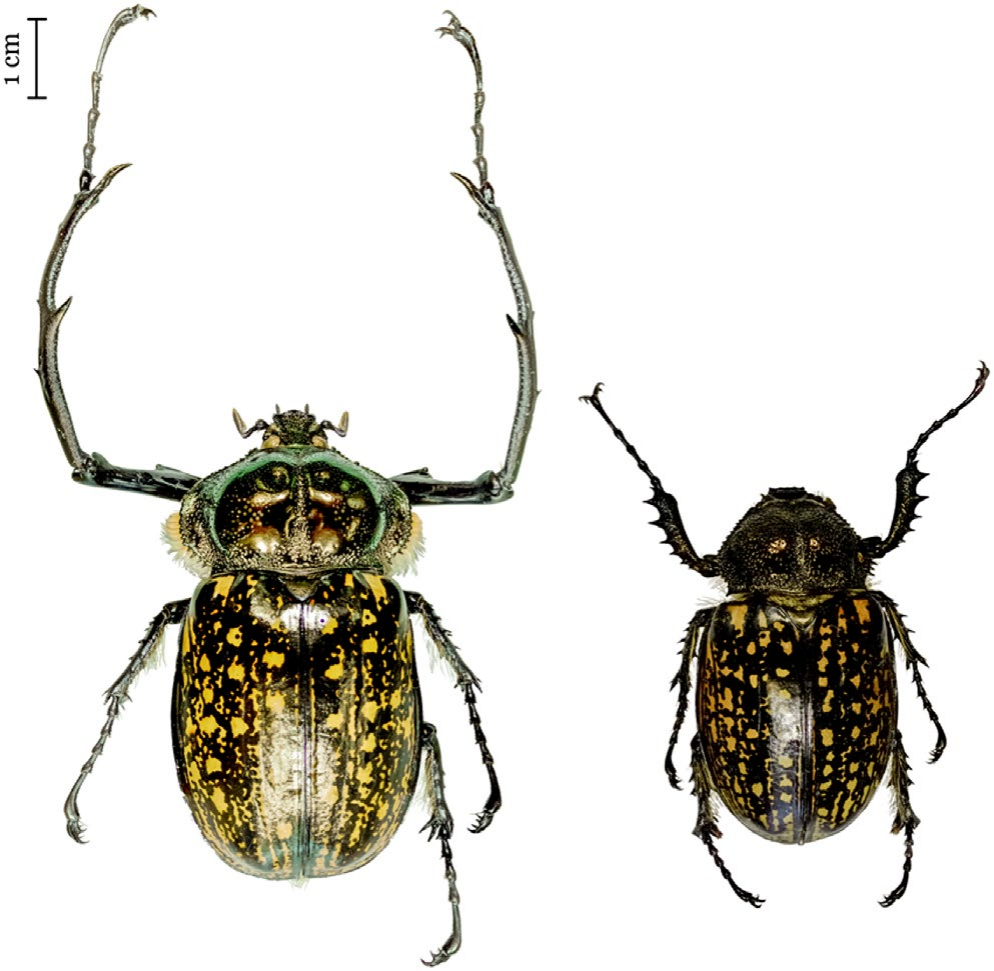
Adult *Cheirotonus formosanus*. Male (left) and female (right). The male's left hind leg was removed for DNA extraction. Adults have metallic dark‐brown elytra with scattered light‐brown spots and golden hairs covering the ventral of the thorax and abdomen. Body length ranges from ~50–65 mm and males exhibit exaggerated, elongated forelegs.

The long‐armed scarabs are large beetles (Coleoptera: Scarabaeidae) that exhibit exaggerated male secondary structures and charismatic body coloration and patterns (Figure 1). The genus *Cheirotonus* includes 8 described species ranging from the Japanese Okinawa south to Indonesian Sulawesi and southeast Asia and across mainland Southeast Asia into Himalayan foothills. Two species are island endemic (Taiwan and Okinawa). The forearms of adult males serve as weapons in male–male competition, and this trait has diversified through changes in the length, shape, and number of spikes on the forearm that differ among species (Young 1989). Additionally, the long‐armed scarab possesses a unique feature among scarab beetles, which is an additional pair of hooks that has evolved on the claws. The function of this extra pair of hooks, however, remains to be studied. In addition to these evolutionary novelties, due to the rising collection pressure on showy scarab beetles (Huang et al. 2024; Young 1989), many local governments have listed the long‐armed scarab as a protected species (Huang et al. 2024; Yu et al. 2025). Consequently, a reference genome would not only facilitate comparative genomic studies to understand the evolutionary origins of phenotypic novelties in these beetles but also enable objective evaluation of the impacts of historically documented anthropogenic changes and current protection policies on their genetic diversity and prospects for long‐term survival.

In this study, we present a high‐quality, chromosome‐level reference genome for the endangered, endemic Formosan long‐armed scarab beetle (*Cheirotonus formosanus*; Figure 1), a species strictly protected in Taiwan since 1989 (Huang et al. 2024). The assembly resolves the sex chromosomes, identifying an X‐linked and a Y‐linked scaffold, and includes a complete circular mitochondrial genome with its repetitive control region. Leveraging these resources, we reconstructed long‐term *N*_*e*_ from single individual genomes (a male and a female, respectively) and estimated recent *N*_*e*_ from published ddRAD‐seq data. Previous genome‐wide SNP data revealed that the species has maintained a relatively stable *N*_*e*_ since the Holocene (Huang et al. 2024). Although there was evidence of geographic association in population genetic differences, the Formosan long‐armed scarab beetle generally exhibits a single genetic population (*K* = 1) within Taiwan (Huang et al. 2024). Nevertheless, despite the lack of apparent geographical genetic structure, individuals within the species show signs of genetic inbreeding (Huang et al. 2024). This suggests that there may be hidden forces not detectable using genome‐wide SNP data that have led to the observed counterintuitive patterns. A high‐quality reference genome, which provides additional information such as linkage among sequenced loci, may help extract additional information from genome‐wide polymorphism data and allow finer temporal‐scale inferences of demographic dynamics critical for biodiversity conservation (Shen et al. 2025). These resources provide a foundation for population genomics and conservation of *C. formosanus*. Beyond this species, these data provide a reference framework for beetle Y‐chromosome characterization and a high‐quality mitogenome assembly for comparative work.

## 2 Materials and Methods

### 2.1 Sample Collection

We collected adult beetles using light traps in Taitung, Taiwan. Larvae used for RNA sequencing were obtained from the offspring of these adult beetles. Two adult beetles were shipped to Texas A&M University (College Station, Texas, USA), with samples kept in −80°C or dry ice. Export of the specimens was authorized by the Ministry of Agriculture of Taiwan under the Wildlife and Wildlife Products Export Permit (Permit No. AGF4X112080011) and USDA (letter of non‐jurisdictions), and importation was declared to the U.S. Fish and Wildlife Service (Confirmation# 2023HN3206664). All samples were stored at −80°C until extraction.

### 2.2 DNA, RNA, and Hi‐C Isolation

High molecular weight DNA extraction and sequencing were performed by Maryland Genomics at the Institute for Genome Sciences, University of Maryland School of Medicine. Approximately 30 mg of leg tissue was used for extraction with the Nanobind PanDNA Kit. A library was constructed with SMRTbell Prep Kit 3.0. Extraction and library preparation were performed according to the manufacturer's protocol and the DNA was then sequenced with Revio SMRT cell (25 M) on the Revio instrument (Pacific Biosciences, Menlo Park, CA). We used DNeasy Blood & Tissue Kit (Qiagen) to isolate DNA from a female adult beetle and TRIzol Reagent with the PureLink RNA Mini Kit with modified manufacturer's protocol to isolate RNA from larvae, and the libraries were prepared and sequenced on the Illumina 150 bp paired end by Genomics BioSci & Tech (Taipei, Taiwan). Hi‐C library prep was performed by CD genomics (Shirley, NY, USA). Hi‐C libraries were constructed according to previous studies (Padmarasu et al. 2019) and the ligated DNA was extracted through QIAamp DNA Mini Kit (Qiagen) according to manufacturers' instructions. The Hi‐C libraries were quantified and sequenced on the Illumina Nova‐seq platform (San Diego, CA, USA).

### 2.3 Genome Size Estimate & Genome Assembly

PacBio HiFi reads were processed using HiFiAdapterFilt (v3.0.1) with the default parameters to remove adapter sequences (Sim et al. 2022). To estimate the genome size, we used a k‐mer‐based approach using Jellyfish (v2.2.10) with a k‐mer size of 21 (Marçais and Kingsford 2011). The k‐mer frequency distribution was analyzed by GenomeScope to infer the genome size, heterozygosity and coverage (Ranallo‐Benavidez et al. 2020). Genome assembly was performed using HiFiasm (v0.24.0) with default setting, integrating PacBio HiFi reads with Hi‐C paired end reads to get an initial contig level assembly (Cheng et al. 2021). We screened the contig level assembly for contamination sequences using NCBI's Foreign Contamination Screen (FCS‐GX) (O'Leary et al. 2024) We downloaded the FCS‐GX database (accessed September 22, 2025) and ran the *fcs.py* script with the target taxon set to NCBI TaxID 7055, which represents the family of Scarabaeidae. We then mapped HiFi raw reads to the contig level assembly and filtered out alignments with mapping quality lower than 30, and calculated the read depth for each locus to access the distribution across contigs. Contigs classified outside Scarabaeidae and showing abnormally low read depth were removed prior to scaffolding. For the scaffolding process, we integrated Omni‐C's mapping pipeline (https://dovetail‐analysis.readthedocs.io/en/latest/). The retained contigs were used as input, and Hi‐C reads were aligned to the contig assembly using bwa‐mem (v2.2.1) with −5SP ‐T0 parameters (Li and Durbin 2009). The mapped reads were filtered by pairtools (v1.1.2) (Open2C et al. 2023). Only mapped reads that had mapping quality over 40 and the first 5 uniquely mapped alignments were kept and alignment gap was set within 30 bp. PCR duplicates were marked and removed. The filtered alignment file was sorted and indexed by samtools (v1.21) (Li et al. 2009). Last, scaffolding was performed by YaHS pipeline (v1.1.2) with default parameters, using filtered BAM files and the initial contig assembly as inputs (Zhou et al. 2023). The final scaffolded assembly was examined by BUSCO (v6.0.0) with the lineage endopterygota_odb10 dataset (Manni et al. 2021). A Hi‐C contact heatmap was generated by the HapHiC plot function and visually inspected to assess the assembly for potential misassembly (Zeng et al. 2024).

The mitochondrial genome was assembled using MitoHiFi (v3.2.1) (Uliano‐Silva et al. 2023). To select an appropriate reference, we used the pipeline's *findMitoRefernce.py* script to download a closely related mitogenome from NCBI ( *C. jansoni* ; FASTA and GenBank format; accession #: NC_023246). We then ran MitoHiFi pipeline with the reads argument (mitohifi.py ‐r) from the raw PacBio HiFi reads to identify mitochondrial reads and assembled and annotated them. To assess mitochondrial genome collinearity, we compared our assembly to closely related mitogenomes. We first rotates all sequences to a common start position using Circlator (v1.5.5) (Hunt et al. 2015) fixstart function (default setting), then performed pairwise BLASTN alignments (BLAST+ v2.16.0; blastn ‐evalue 1e‐2 ‐word_size 7 ‐dust no ‐gapopen 2 ‐gapextend 2 ‐reward 1 ‐penalty −1 ‐outfmt 6) (Camacho et al. 2009). BLAST hits were plotted in R to visualize alignment coverage and identify along the circular genome.

### 2.4 Repetitive Content & Genome Annotation

RepeatModeler (v2.0.5) was used to identify and model *de novo* transposable elements (TEs) from the scaffolded genome assembly (Flynn et al. 2020). Additionally, a Coleoptera‐specific repeat library (Taxon ID: 7041) was extracted from the RepeatModeler database. These two libraries were combined to create a custom repeat library. The scaffold genome was soft‐masked using Repeatmasker (v4.1.7) with the custom library with slow search mode and rmblast as the search engine (Tarailo‐Graovac and Chen 2009).

RNA reads were aligned to the final scaffolded genome using STAR (v2.7.10b), which is a splice‐aware aligner (Dobin et al. 2013). The RNA alignments and soft‐masking genome, along with Arthropoda protein database from OrthoDB v12 were provided as input to train GeneMark‐ETP and AUGUSTUS in the BRAKER3 pipeline (v3.0.7) (Brůna et al. 2021; Bruna et al. 2024; Buchfink et al. 2015; Gabriel et al. 2021, 2023; Gotoh 2008; Hoff et al. 2016, 2019; Iwata and Gotoh 2012; Kovaka et al. 2019; Pertea and Pertea 2020; Stanke et al. 2006, 2008). To produce non‐redundant gene models, isoforms from the BRAKER3 output (*braker.aa*) were removed by a custom script *remove_isoforms.sh* (https://github.com/seanchien4/genome‐assembly‐pipeline). This script retains the longest isoform from each gene; if multiple isoforms share the same length, the first isoform is retained. The final gene set was assessed for completeness using BUSCO (v6.0.0) with the lineage endopterygota_odb10 dataset (Manni et al. 2021). Then, Interproscan (v5.60) was used to predict the function for each gene (Zdobnov and Apweiler 2001).

### 2.5 Identify Sex Linked Chromosomes

To identify sex specific chromosomes, we used the chromosome‐quotient approach, which compares normalized read depth between males and females to distinguish autosomes, X‐linked scaffolds, and Y‐linked scaffolds. We first mapped male reads (PacBio HiFi) and female reads (Illumina PE150) to the final scaffold assembly and filtered out reads with mapping quality < 30 using minimap2 (v2.28) and samtools (v1.21) (Li 2018; Li et al. 2009). We then output and plotted both male and female read depth for each locus, respectively. Because per‐base coverage is skewed by outliers (very low read depth from sequencing/mapping errors or contaminations and very high depths from repeats/collapsed duplication), we used modal depth for each sex as a robust estimate of single‐copy coverage rather than the mean. Using a custom python script, we normalized the read depth at each locus by dividing by the sex specific mode read depth. Loci with 0 read depth in males were marked as DQ (disqualified) because male reads are expected to map to all scaffolds. Loci with 0 read depth in females were retained, as they may represent male‐specific regions. Loci with normalized read depth > 3 were also marked as DQ, as high read depth suggests mapping errors or repetitive regions that were not fully resolved. Once male and female normalized read depths were obtained for each locus, we used another custom python script to calculate the average female to male read depth ratio across defined window sizes (5 bp) and plotted the density of their ratios. From the density plot of normalized female to male read depth ratios for scaffold, scaffolds were classified as autosomes, X‐linked, or Y‐linked based on ratios.

### 2.6 Demographic History & Divergence Time

We inferred historical *N*_*e*_ with PSMC pipeline using female Illumina whole‐genome reads and a male PacBio HiFi reads. Reads were mapped to the 9 largest autosomal scaffolds from the scaffold level assembly (excluding sex‐linked scaffolds and small scaffolds with noisy sex bias signals) with minimap2 v2.28 (Illumina: ‐x sr; PacBio HiFi: ‐x map‐hifi), and alignments were sorted with duplicates marked using GATK MarkDuplicates v4.5.0.0 (McKenna et al. 2010). We retained mapped, properly paired reads with MAPQ ≥ 30. Genotype likelihoods were computed and variants called with BCFtools v1.19 with base‐quality threshold Q ≥ 20 by bcftools call (bcftools mpileup ‐Q 20 | bcftools call). A diploid consensus in FASTQ format was generated from the VCF (bcftools consensus) and converted to PSMC input using *fq2psmcfa* (−q20). We split the consensus with *splitfa* and ran PSMC with standard parameters, performing 100 bootstrap resampling of split segments to assess uncertainty. Bootstrap outputs were concatenated and plotted; trajectories were scaled using a mutation rate of 5.8 × 10^−9^ per site per generation and a generation time of 2 years (Xu et al. 2026).

We inferred recent *N*_*e*_ from published ddRAD seq data using GONE2 (v2.0) (Santiago et al. 2025) (Accession: PRJNA1037179, *n* = 46, (Huang et al. 2024)). ddRAD reads were mapped to the 9 largest autosomal scaffolds of the scaffold level assembly with BWA‐mem2 (v.2.2.1) default setting (Jung and Han 2022), and alignments were filtered to retain mapped reads with MAPQ ≥ 30 using samtools (v1.21) (Li and Durbin 2009). Variants were called with the GATK pipeline. Per‐sample gVCFs were generated with HaplotypeCaller (NVIDIA Parabrick v4.5.0), merged with CombineGVCFs v.4.5.0.0, and jointly genotyped with GenotypeGVCFs. To handle missing data and low coverage typical of ddRAD, we applied a two stage filtering pipeline. First, we generated a prefiltered VCF by removing indels and retaining sites with QUAL ≥ 30(vcftools ‐remove‐indels ‐minQ 30). We then calculated per‐individuals missingness and ranked samples accordingly and created four subsamples consisting of the top 10, 20, 30, and 40 individuals with the lowest missingness. For each subsample, we applied final (second stage) filters: retaining only biallelic SNPs, removing indels, and enforcing a site‐mean depth of 10–100 and per‐genotype depth of 10–100 (vcftools ‐keep include. list ‐remove‐indels ‐min‐alleles 2 ‐max‐alleles 2 ‐minQ 20 ‐min‐meanDP 10 ‐max‐meanDP 100 ‐minDP 10 ‐maxDP 100). The filtered VCF was converted to PLINK format (.ped/.map) with PLINK v2.00 with a strictly zero‐missingness threshold (‐geno 0.0), and GONE2 (v2.0) was run 100 independent times for each subset, assuming a constant recombination rate of 1.3 cM/Mb across the genome to estimate recent *N*_*e*_ change (Lorenzen et al. 2005). We summarized the demographic trajectories by calculating the median *Ne* and the 95% confidence intervals (quantiles 0.025 and 0.975) across all 100 iterations.

To further characterize the recent demographic history and distinguish between historical bottlenecks and recent inbreeding, we identified runs of homozygosity (ROH) using the chromosome‐level assembly and the filtered ddRAD seq dataset. Variants were filtered using VCFtools (v0.1.16) to retain only biallelic SNPs with a minimum quality score of 30, a maximum of 20% missing data per site, and to remove indels (vcftools: ‐remove‐indels ‐min‐alleles 2 ‐max‐alleles 2 ‐max‐missing 0.8 ‐minQ 30). We used the hidden Markov model approach implemented in BCFtools (v1.19) (bcftools roh) (Narasimhan et al. 2016) to account for the reduced‐representation nature of the data. Allele frequencies were estimated directly from the 46 samples, and the resulting genomic regions of homozygosity were extracted for downstream analysis of ROH length categories and total genomic coverage.

Phylogenetic dating analyses were performed in BEAST2 (v2.7.8) (Bouckaert et al. 2019) using a concatenated alignment of the 13 mitochondrial coding sequence (CDS) from 6 taxa: 3 focal *Cheirotonus* species (including *C. formosanus* from this study and published mitogenomes for *C. jansoni* , NC_023246.1, and *C. gestroi* , NC_046890.1), two representatives of the closely related subfamily Euchirinae (*Euchirus longimanus*, OR253996.1; *Propomacrus bimucronatus*, NC_070352.1), and an outgroup from Lucanidae (*Lucanus cervus*, MK059422.1). Individual CDSs were extracted, aligned with MAFFT v7.526 (default settings), visually inspected and edited in Seqotron, and concatenated into a supermatrix (Katoh et al. 2002). To obtain a starting topology, we ran RAxML (v8.2.12) (model GTRGAMMA; −f a ‐m GTRGAMMA ‐p 12,345 ‐x 12,345 ‐# 100) with *L. cervus* as outgroup and used the best scoring ML tree as starting tree in BEAST2. In BEAST2, we implemented a GTR substitution model with no among‐site rate variation, a strict molecular clock model, and birth‐death tree prior were implemented. Divergence times were calibrated using a normal prior on the root node, based on published estimates for the split between Family Scarabaeidae and Family Lucanidae (mean = 183.79 million years ago (MYA), standard deviation = 13.99 MYA) (D. D. McKenna et al. 2019). All other parameters were left at default settings. Markov chain Monte Carlo runs were 10 million generations, sampled at 1000 generation intervals; convergence was assessed in Tracer (ESS > 200). We discarded the first 50% of MCMC samples as burn‐in and summarized a maximum clade credibility (MCC) tree in TreeAnnotator (BEAST2 v2.7.8), specifying node height as mean. Divergence times were estimated as the age of the most recent common ancestor (MRCA) for the specified taxa and we reported the mean, median, and 95% posterior density intervals of MRCA ages.

## 3 Results

### 3.1 Genome Size Estimate & Genome Assembly

**FIGURE 2:**
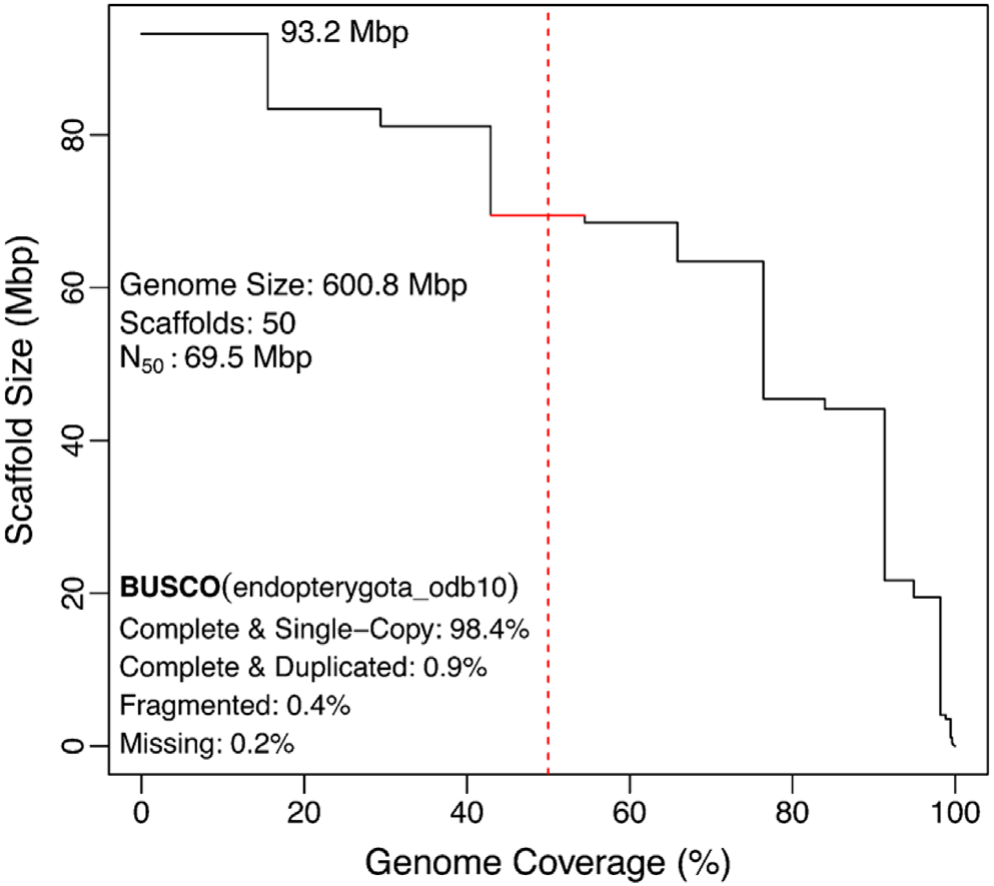
*Cheirotonus formosanus* Genome assembly contiguity and completeness. Scaffolds are ordered by descending length. The x‐axis shows the cumulative percentage of the genome accounted for as scaffolds are added, and the y‐axis shows scaffold length. The largest scaffold is 93.2 Mbp, and the *N*_50_ is 69.5 Mbp, reaching the 4th scaffold. BUSCO completeness is 99.3% (98.4% single‐copy BUSCOs, 0.9% duplicated BUSCOs, 0.4% fragmented, and 0.2% missing).

**FIGURE 3:**
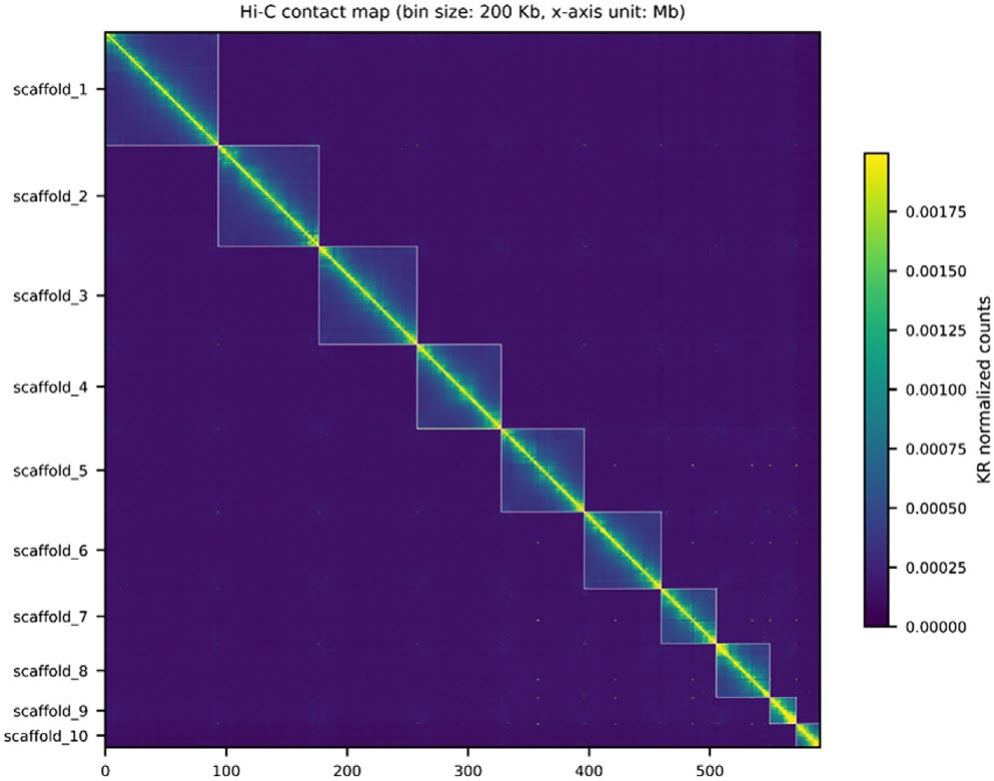
Hi‐C contact heatmap of *Cheirotonus formosanus* genome. The heatmap shows normalized interaction frequency among scaffolds (bin size 200 kbp). Scaffolds are ordered and oriented according to Hi‐C scaffolding. Square blocks along the diagonal indicate chromosome‐level contiguity.

Using a k‐mer size 21, the k‐mer distribution showed a clear homozygous peak at the depth of 58. Based on this histogram, GenomeScope estimated the genome size at approximately 492 Mbp with heterozygosity 1.06% (Figure S1). The initial contig level genome assembly consisted of 62 contigs with a total genome size of 600.9 Mbp and *N*_50_ of 69.5 Mbp. FCS pipeline identified 10 contigs as putative contaminants (Table S1). In the read depth analysis, these contigs showed significantly lower coverage compared to the majority of contigs and were therefore removed from further analyses (Figure S2). After scaffolding with the YaHS scaffolding pipeline, the assembly contained 50 scaffolds with an *N*_50_ of 69.5 Mbp. The initial genome assembly contained near chromosome level contigs, Hi‐C scaffolding mainly ordered contigs and improved small fragments, with little effect on *N*_50_. The largest scaffold measured 93.2 Mbp and 10 scaffolds exceed 15Mbp in size, together covering 98.2% of the assembled genome (Figure 2; Figure 3). Genome completeness was assessed using BUSCO (v6.0.0) with the endeopterygota_odb10 lineage dataset (2124 BUSCOs). The result indicated 99.3% completeness, including 98.4% single‐copy BUSCOs, 0.9% duplicated BUSCOs, 0.4% fragmented, and 0.2% missing. Together, these results demonstrate that the assembly captures nearly the full gene content and represents a highly complete genome.

The mitochondrial genome was identified and annotated using MitoHiFi (v3.2.1). The *C. formosanus* mitochondrial genome is 20,286 bp in length with GC content of 30.74% and contains the canonical set of 37 genes (22 tRNAs, 2 rRNAs, and 13 protein coding genes).

### 3.2 Repetitive Content & Genome Annotation

**FIGURE 4:**
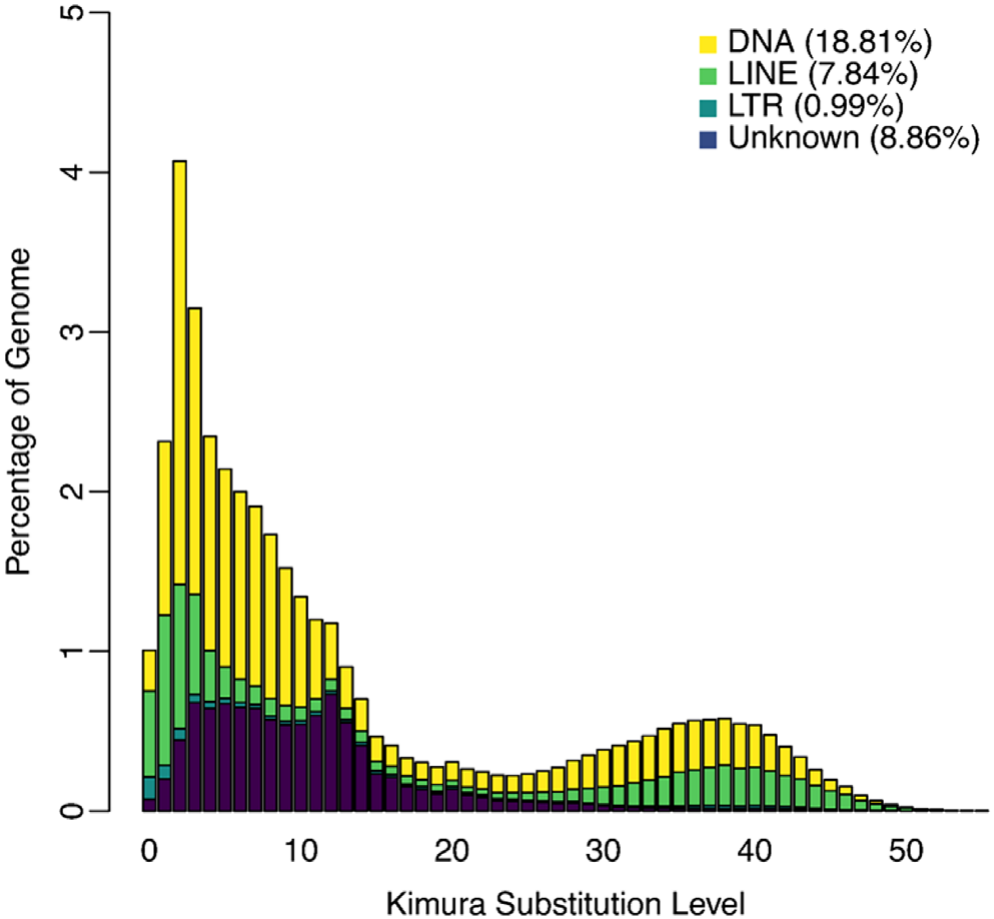
Repeat landscape plot of the *Cheirotonus formosanus* genome. The plot shows the distribution of transposable element (TE) copies annotated by Repeatmasker across Kimura two‐parameter (K2P) divergence to their consensus sequence.

Using both a *de novo* TE library and lineage specific (Coleoptera) repeat library, RepeatMasker identified and annotated 37.81% of the scaffolded genome as repetitive sequences. These include 18.81% DNA transposons, 8.82% retroelements, and smaller fractions of simple repeats (1.00%) and low complexity (0.23%) regions. In addition, 8.86% of repeats remained unclassified (Figure 4). The BRAKER3 genome annotation pipeline identified 12,736 protein coding sequences in the final gene set (braker.gtf). Genome annotation completeness was assessed using BUSCO (v6.0.0) with the endeopterygota_odb10 lineage dataset (2124 BUSCOs). After removing the isoforms from the dataset, the duplication rate was reduced from 20.0% to 1.5% (96.2% single‐copy BUSCOs, 1.5% duplicated BUSCOs, 0.3% fragmented, and 2.0% missing).

### 3.3 Identify Sex Linked Chromosomes

**FIGURE 5:**
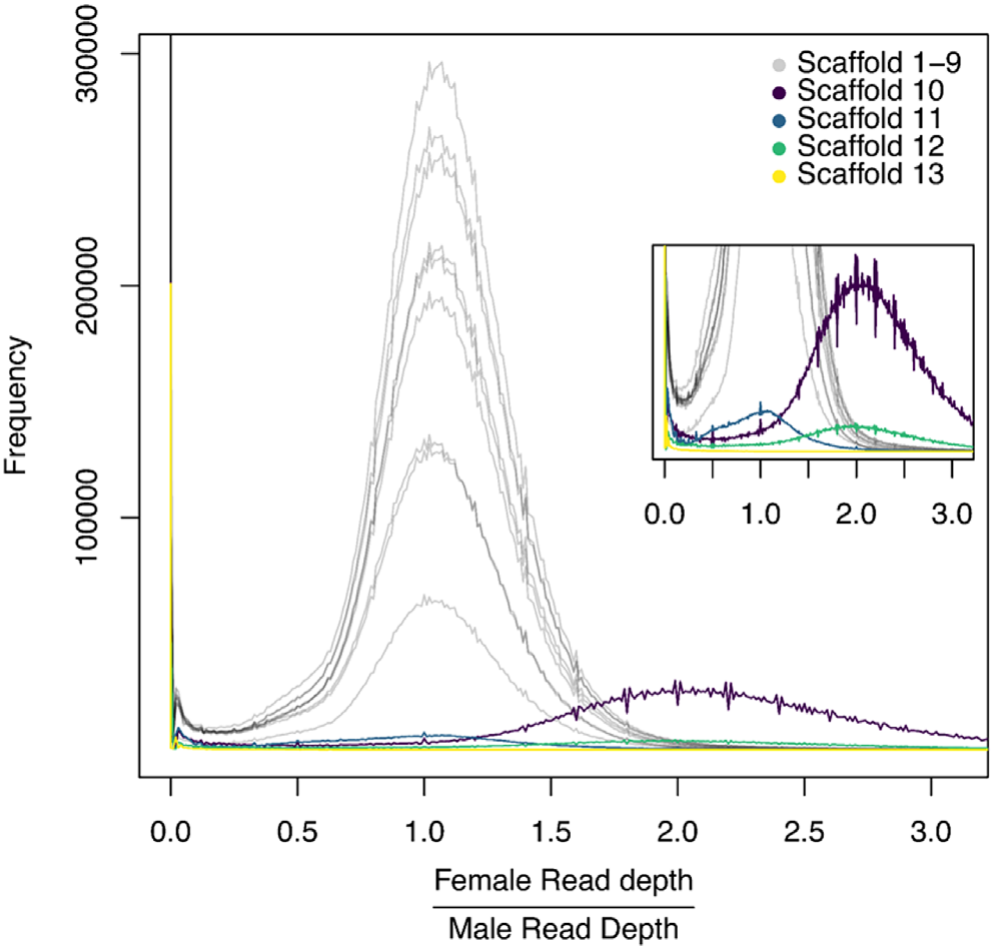
Normalized female: Male average read depth ratios in fixed window (5 bp) for each scaffold over 1 Mbp. Ratios near 1 indicated autosome scaffold; ratios near 2 indicate. X‐linked scaffolds (females carry two copies); ratios near 0 indicate Y‐linked scaffold (absent in females).

Using the locus level read depth density plots, the genome wide modal read depths were ~60× and~65× for the female and male, respectively (Figure S3). Scaffolds < 1 Mbp showed highly variable female: male depth ratios, likely due to low coverage and assembly artifacts, so we restricted chromosome assignment to scaffolds ≥ 1 Mbp (*n* = 13). For each scaffold, we computed the normalized coverage ratio (per locus depth divided by the sex specific modal depth) and average ratios in a fixed window (5 bp). The 9 largest scaffolds had ratios centred near 1.0, consistent with autosomes (equal copy number in both sexes). The 10th scaffold had a ratio centered near 2.0, consistent with the X chromosome (females carry an extra copy). The 11th and 12th scaffold (4.1 Mbp & 3.5 Mbp) showed ratios consistent with an autosome and an X‐linked fragment, respectively, and likely represent sequences that failed to scaffold with larger scaffolds. The 13th scaffold (1.1 Mbp) exhibited a high frequency of ratios near 0 and was classified as Y‐linked (Figure 5). The male normalized depth on this scaffold averaged ~0.5×, consistent with hemizygous, single‐copy Y sequence in males (Figure S3).

### 3.4 Demographic History & Divergence Time

**FIGURE 6:**
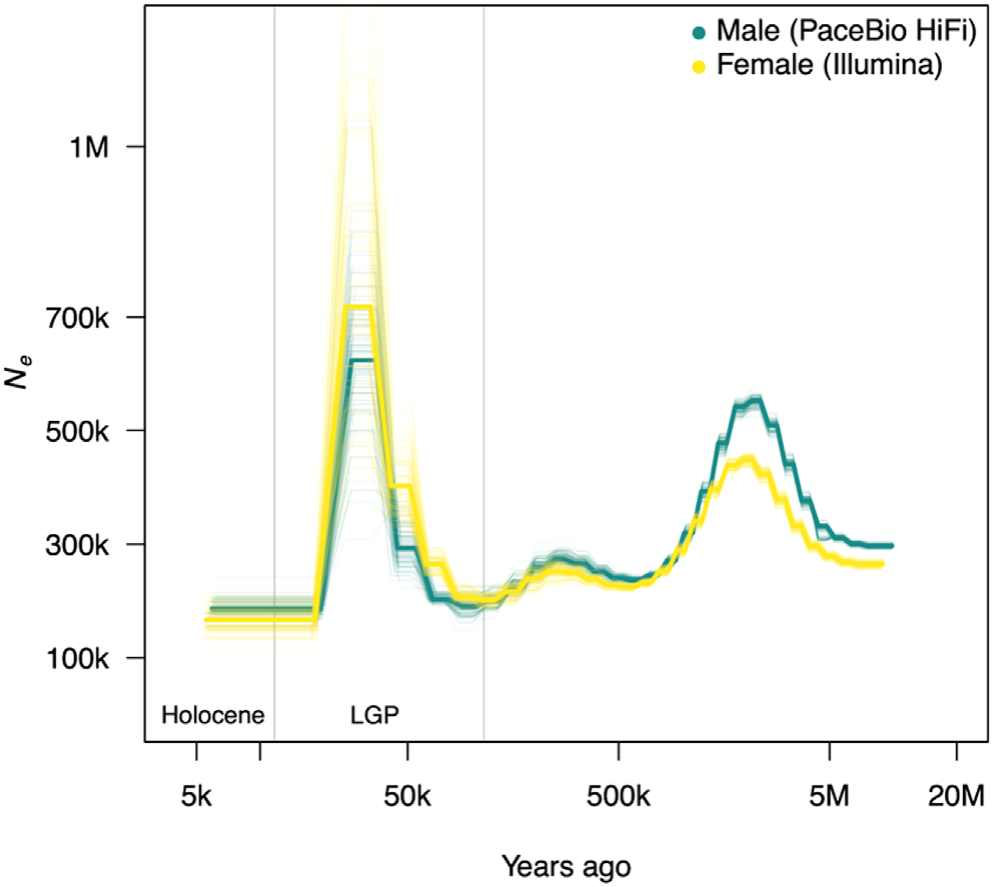
Historical *N*_*e*_ inferred with PSMC from male (PacBio) and female (Illimina PE150) genomes. Faint lines represent bootstrap replicates and solid lines represent the best run. The x‐axis is time 10^3^ years ago before present; the y‐axis is *N*_*e*_. Estimates were scaled using a mutation rate of 5.8 × 10^−9^ per site per generation and a generation time of 2 years.

**FIGURE 7:**
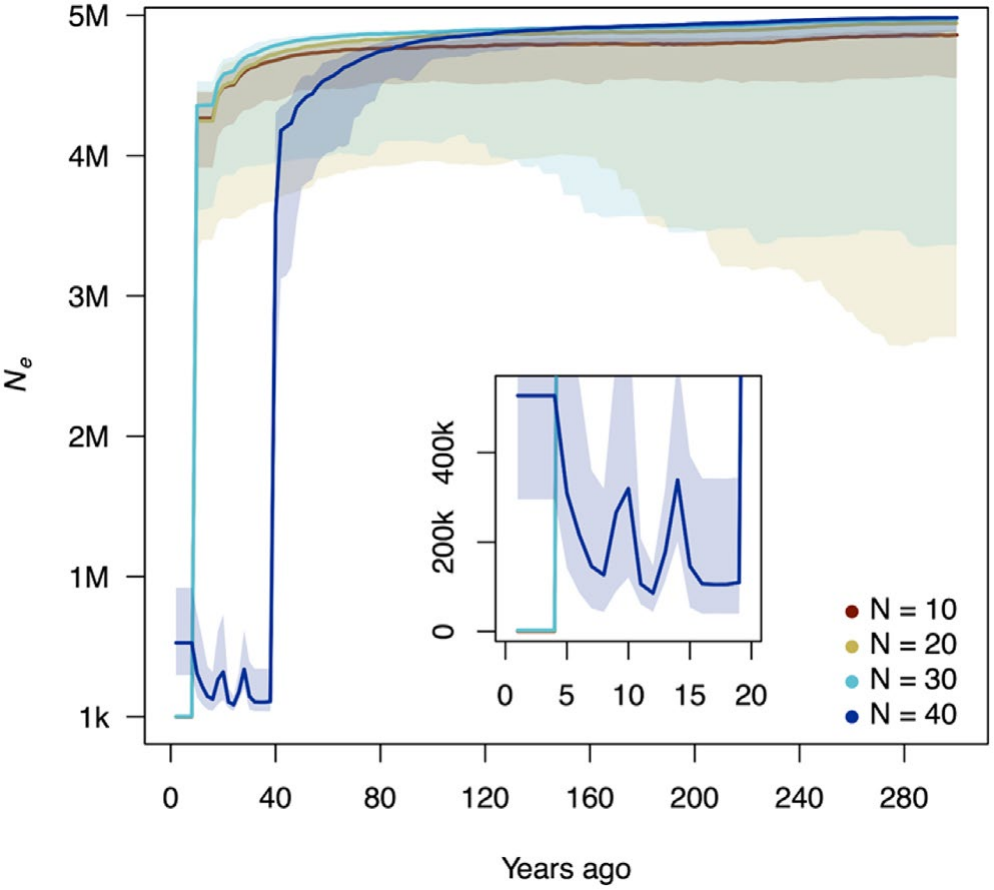
Recent *N*_*e*_ inferred with GONE2 from published ddRAD seq data. Colored lines represent estimates using subsamples of 10, 20, 30, and 40 individuals. Shaded areas surrounding each median line represent the 95% confidence intervals (0.025 and 0.975 quantiles) calculated from 100 independent iterations per subsample. The x‐axis is years before present and the y‐axis is *N*_*e*_. Analyses assumed a constant recombination rate of 1.3 cM.

We reconstructed past *N*_*e*_ from two *C. formosanus* genomes (male: PacBio HiFi; female: Illumina). The two trajectories are nearly identical and their bootstrap overlap, indicating that the signal is robust across sequencing platforms. *N*_*e*_ was moderate for much of the last 500 thousand years ago (kya) (Figure 6). Beginning around 115 kya, *N*_*e*_ rose and then increased sharply to a short peak around 50 kya. *N*_*e*_ subsequently declined steeply toward the Last Glacial Period and remained approximately constant through the Holocene (Figure 6). Very recent changes (< 10 kya) are not resolved by PSMC. In contrast, GONE2 inferred broadly consistent *N*_*e*_ across subsamples of 10, 20, 30, and 40 individuals, with 63,592, 43,116, 22,417, 5416 SNPs, respectively. All subsamples have a constant *N*_*e*_ across 200–300 years ago. Approaching the present, *N*_*e*_ becomes unstable and shows an apparent decline in the last 50 years with a slight increase in the last 20 years in the *N* = 40 (Figure 7).

**FIGURE 8:**
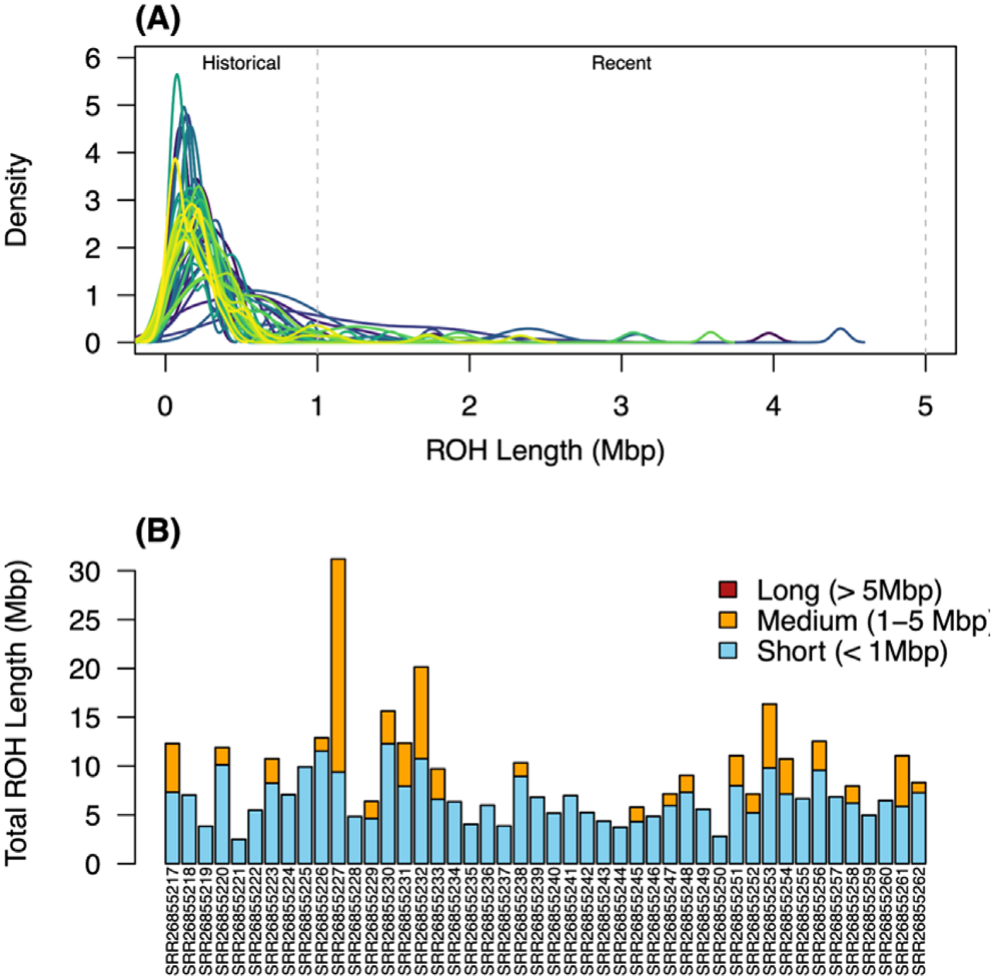
Runs of Homozygosity (ROH) profile from published ddRAD seq data. (A) Density distribution of ROH segment lengths for 46 individuals. Each colored line represents an individual genome. The distribution is characterized by a high density of short segments (< 1 Mbp), reflecting historical bottlenecks or long‐term persistence at a small *N*_*e*_. (B) Cumulative length of ROH per individual, categorized by segment size. Bars represent total genomic coverage of short (< 1 Mbp), medium (1–5 Mbp), and long (> 5 Mbp) ROH segments. The dominance of short segments and the total absence of segments exceeding 5 Mbp suggest that current inbreeding levels are driven by historical demographic restriction rather than extreme recent inbreeding.

Analysis of ROH revealed a landscape dominated by short segments (< 1 Mbp), a pattern characteristic of historical bottlenecks or long‐term persistence at a low ancestral *N*_*e*_ (Figure 8). While the majority of the genomic homozygosity is comprised of these shorter fragments, we identified medium‐length segments (1–5 Mbp) in several individuals, indicating recent inbreeding events within localized demes. Notably, we found a total absence of very long segments (> 5 Mbp) (Figure 8). This suggests that while the population may be small and historically restricted, recent inbreeding (e.g., within the last few generations) does not appear to be widespread in the current breeding pool.

The MCC tree indicates that *C. formosanus* is more closely related to *C. gestroi* than *C. jansoni* . The posterior mean MRCA age for *C. formosanus* and *C. gestroi* was 38.2 MYA (95% HPD: 32.1–44.8 MYA). The divergence between *C. jansoni* and *C. formosanus* and *C. gestroi* was 106.47 MYA (95% HPD: 89.7–123.2 MYA) (Figure S4).

## 4 Discussion

Although the contig and scaffold level assembly were similar between the long‐read only assembly and the Hi‐C integrated assembly, Hi‐C data were critical for identifying and correcting misassemblies and for achieving robust chromosomal level assembly. In our initial long read‐only hifiasm (v0.19.9) assembly, Hi‐C contact maps revealed at least one major misjoin in the largest contig (126.4 Mbp). Interactive review and manual breaking followed by re‐scaffolding resolved the inconsistency. The final corrected contact map displayed 10 primary large scaffolds, including 9 autosomes and X chromosomes. This genetic architecture is highly consistent with known cytogenetic data for the group. The majority of Coleoptera possess a diploid number of 2*n* = 20 (9AA + XY) (Blackmon and Demuth 2015). Furthermore, members of the related subfamily Euchirinae (e.g., *Propomacrus*) have been reported with a consistent 2*n* = 20 karyotype (Dutrillaux and Dutrillaux 2012). The high degree of synteny and the recovery of 10 major scaffolds in our Hi‐C assembly provide strong evidence that these scaffolds represent the actual physical chromosomes of the species.

### 4.1 Mitochondria Genomes

**FIGURE 9:**
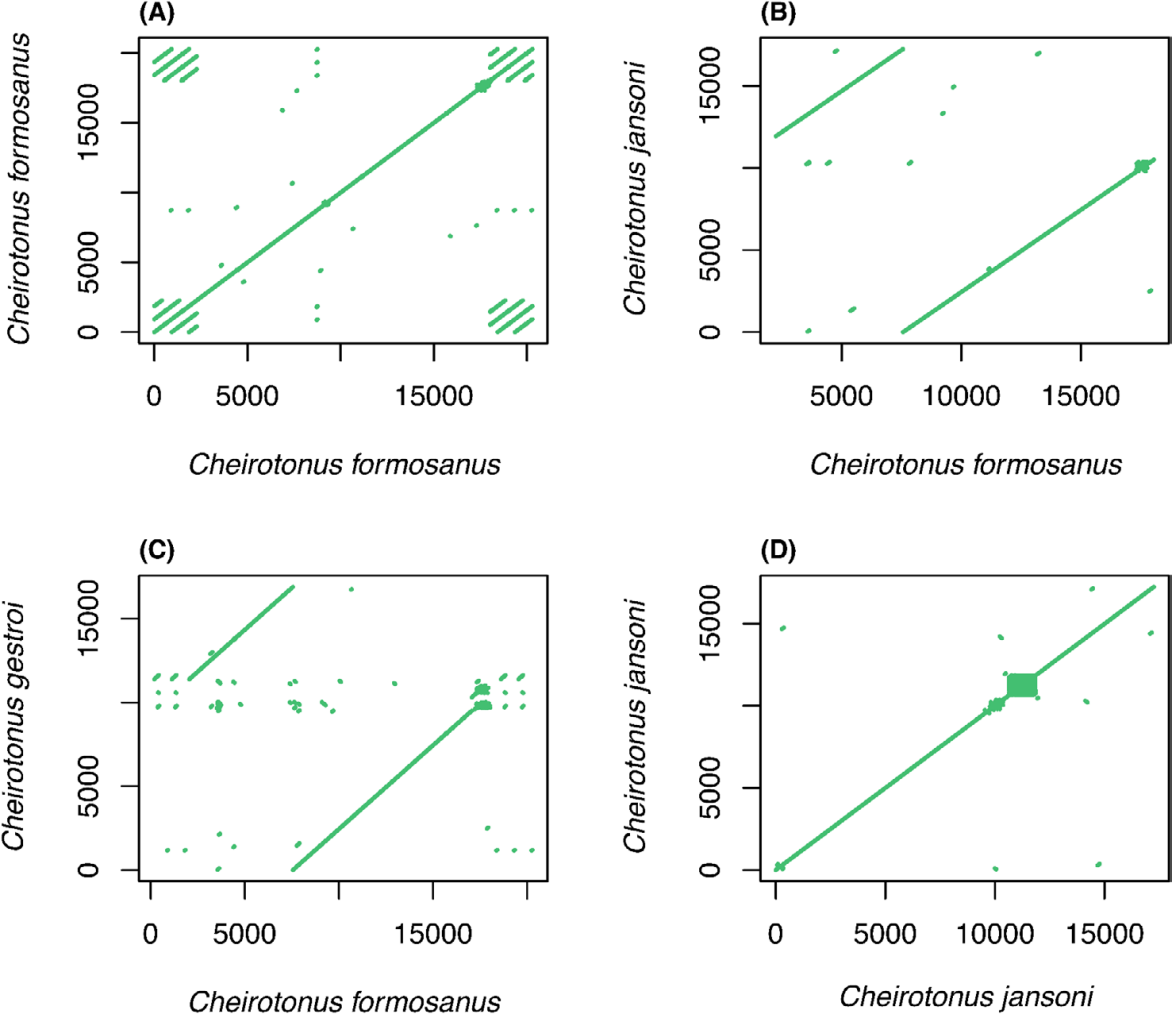
Pairwise BLASTN comparisons of mitochondrial genome. We rotated all genome to a common start using Circlator default setting. (A) *Cheirotonus formosanus* versus *C. formosanus* (self‐alignment): a continuous end to start match with duplicated hits at the junction indicates circularity and tandem repeats in the control region. (B and C) *C. formosanus* versus *C. jansoni* and *C. formosanus* versus *C. gestroi*: high collinearity across coding regions with fragmented hits in the control region consistent with unresolved or missing repeats in the short‐read assemblies. (D) *C. jansoni* versus *C. jansoni* (self‐alignment): a collapsed repeat cluster at the circular junction indicates that short‐read data did not resolve the control region tandem repeats.

MitoHiFi recovered a complete circular mitochondrial genome for *C. formosanus*. All 37 genes are present and long reads provide continuous coverage across the circular junction. The assembled mitochondrial genome is longer than those of two closely related species (*C. jansoni & C. gestroi*) with the length difference concentrated in the repetitive regions (Figure 9). Our BLASTN analysis confirms that these additional sequences consist of tandem repeat units within the control region, suggesting that the previously reported shorter lengths in these related species likely resulted from collapsed assemblies of these repetitive arrays. This is consistent with the findings that long reads reconstruct tandem repeat arrays and structural variation in the control region that short‐read assemblies collapse or truncate (Morgan et al. 2022). Together, these results illustrate how long read approaches resolve repetitive mitochondrial regions that short‐read assemblies often leave incomplete, improving comparative and phylogenetic utility.

### 4.2 Identify Sex Linked Chromosomes

Y chromosomes are typically repeat‐rich and gene poor, which makes them difficult to assemble and anchor confidently. In Polyphaga, the Y is often cytologically punctiform, is fully non‐recombining due to asynaptic meiosis, and is therefore expected to be highly repetitive and small (Blackmon and Demuth 2014). Our candidate Y‐linked scaffold matches these inspections. Long‐read sequencing helps span repeats, and sex biased coverage provides orthogonal evidence for Y linkage. Using the chromosome‐quotient approach, we computed normalized female: male read‐depth ratios in fixed windows (5 bp) across the assembly and identified a 1.1 Mbp scaffold with ratios near 0 across most of its length. The putative Y‐linked scaffold is enriched for repeats and contains 9 gene models annotated by BRAKER3. InterProScan domain annotations indicate one of these models has the *JARID1/KDM5* family architecture. This gene model is covered by male reads but is absent in female data. Because InterProScan reports domain architecture rather than gene names, and because *KDM5* family members have paralogs with sex‐linked distribution in other taxa (e.g., *KDM5D* on the mammalian Y chromosome), we interpret this as KDM5 like demethylase on the Y and recommend additional validation (Kosugi et al. 2020).

Due to the strict conservation status and limited specimen availability of *C. formosanus* , as regulated under the Wildlife and Wildlife Products Export Permit (No. AGF4X112080011) by the Ministry of Agriculture of Taiwan, the number of individuals available for destructive sampling is extremely constrained. While we relied on the chromosome‐quotient method and existing genomic resources for this identification, we were unable to perform broader PCR‐based validation across a larger population panel at this time. As the first whole‐genome resource for this species/genus on NCBI, this assembly provides a critical reference for future studies. Overall, the coverage‐based assignment and gene content support the presence of putative Y‐linked sequence in *C. formosanus*, a rarely characterized feature in beetles, but we note that repeat‐rich regions remain challenging.

### 4.3 Demographic History & Divergence Time

Our PSMC based inference on historical *N*_*e*_, performed independently on male and female genomes, shows a nearly identical long term *N*_*e*_ trajectories and broadly agrees with the ddRAD seq based demographic pattern previously reported (Huang et al. 2024). This consistency indicates that single, high‐quality whole genomes can robustly capture broad, long timescale demographic signals. This is particularly valuable for endangered species where fresh sampling is difficult to obtain. With a reference genome, similar mapping based approaches can also leverage older specimens with fragmented DNA, whereas ddRAD seq requires high‐quality DNA and fresh tissue, which is often challenging for threatened species. For recent *N*_*e*_, we used published ddRAD seq data with GONE2 and inferred *N*_*e*_ across 10–40 individuals (Figure 7). *N*_*e*_ is approximately constant over the last few decades, with an apparent decline in the most recent decades. Because SNP counts decrease markedly as sample size increases (*N* = 10: 63,592; *N* = 20: 43,116; *N* = 30: 22,417; *N* = 40: 5416), reducing information in the near‐present bins, we interpret the near‐present dip as methodologically driven (lower SNP density/missingness and model assumptions) rather than evidence of a documented demographic collapse, and we treat the last few decades cautiously.

We note that a recent drastic decline in *N*_*e*_ may be a true biological pattern. Although such a rapid reduction in *N*_*e*_ is unlikely to generate detectable population subdivision among geographic regions because genetic drift has not had time to fix different alleles across locations (Slatkin 1993; Whitlock and McCauley 1999), a sharp decrease in *N*_*e*_ can produce measurable inbreeding within only a few generations (Keller 2002). However, our data indicates that homozygous tracts are predominantly short in length, suggesting a history of sustained low *N*_*e*_ rather than a recent crash scenario (Figure 8). The absence of tracts exceeding 5 Mbp implies that while the species is genetically impoverished, it has not yet reached the level of extreme inbreeding often seen in rapidly declining populations. This counterintuitive combination (i.e., lack of geographic genetic structure despite clear signals of inbreeding) was documented in a previous study of *C. formosanus* (Huang et al. 2024) and consistent with our results. Therefore, our results may reflect a historical process that can only be uncovered when a high‐quality reference genome is coupled with genome‐wide SNP data (Shen et al. 2025). Ecologically, long‐armed beetles depend on old growth forests because their larvae require specific microhabitats, such as large cavities in mature tree trunks (Young 1989). Although forest cover has been recovering since the 1990s in Taiwan (Chen et al. 2019), much of this regrowth consists of secondary, young forests that may still be unsuitable for supporting a rebound in *N*_*e*_.

Our findings thus have important conservation implications. If we focus on only the apparent panmictic genetic structure of the species (Huang et al. 2024), we may underestimate the consequences of reduced gene flow, while the reality may be that current populations are fragmented into small, inbred demes that were previously undetectable without a reference genome. The species may require active management interventions, such as assisted gene flow, to alleviate the impact of ongoing genetic inbreeding. To determine whether the inferred recent demographic decline reflects a true biological pattern or a model artifact, additional data will be needed. Specifically, sequencing more individuals at higher coverage (i.e., whole‐genome resequencing rather than ddRAD) or incorporating historical samples that provide temporal baselines of genetic diversity (Clark et al. 2025) would strengthen the inference. The current ddRAD dataset likely lacks sufficient coverage to fully exploit linkage information, especially given the large genome size of the beetle. Deeper sequencing would therefore improve model performance. Moreover, adding genomic data from historical specimens would provide empirical datasets for assessing changes in genetic diversity over time. Because this charismatic beetle has been collected globally and preserved in museums over the past century, it is feasible to reconstruct temporal patterns of genetic variation using museum specimens. Importantly, none of these approaches would be feasible without a high‐quality reference genome, which again supports the importance of our genome resource.

The timing and shape of the *N*_*e*_ curve is consistent with regional palaeoclimate for Taiwan. After the Last Interglacial, sea level fell and the Taiwan Strait became progressively shallow, while cooler conditions shifted montane forest belts downslope. Lower sea levels intermittently exposed the continental shelf, narrowing or closing the Taiwan Strait and increasing potential land connectivity with the mainland. At the same time, cooler temperatures shifted montane forest belts downslope, expanding the area of suitable mid‐elevation habitat. As glaciation intensified, colder and likely drier conditions fragmented forests into refugia, and subsequent deglaciation re‐isolated Taiwan as sea level rose and montane habitats shifted upslope. However, the BEAST2 phylogeny from mitochondrial genomes indicates that the divergence time between *C. formosanus* and *C. jestroi* and *C. jansoni* were 38.2 and 106.5 MYA, respectively. This likely overestimates the true separation. Mitochondria DNA is a single, non‐recombining locus, which can produce longer branch lengths due to the rapid fixation of alleles in small or isolated populations. This effect may be particularly common if populations have experienced genetic drift or bottlenecks, leading to rapid lineage sorting. As a result, mitochondrial genomes alone can exaggerate divergence times, and these estimates should be interpreted cautiously (van Tuinen and Torres 2015; Zheng et al. 2011). Analyses of nuclear genomic data can provide a much larger number of independent loci that better capture the true species history (Jansa et al. 2006). Nuclear genomic data with multiple loci will be required to achieve higher phylogenetic resolution and more accurate divergence time estimates for these beetle populations (Wild and Maddison 2008; Zheng and Wiens 2015).

## Author Contributions

**Sean Chien:** conceptualization (equal), data curation (equal), formal analysis (equal), investigation (equal), methodology (equal), validation (equal), visualization (equal), writing – original draft (equal), writing – review and editing (equal). **Jen‐Pan Huang:** conceptualization (equal), data curation (equal), funding acquisition (equal), project administration (equal), writing – review and editing (equal). **Heath Blackmon:** conceptualization (equal), funding acquisition (equal), methodology (equal), project administration (equal), resources (equal), supervision (equal), writing – review and editing (equal).

## Funding

This work was supported by the Taiwanese National Science and Technology Council, NSTC 114‐2621‐B‐001‐006‐MY3. National Institutes of Health (10.13039/100000002), R35GM138098.

## Conflicts of Interest

The authors declare no conflicts of interest.

## Supporting information


**Figure S1:** GenomeScope k‐mer profile from PacBio HiFi reads (*k* = 21). This shows the k‐mer distribution and inferred estimated genome size.
**Figure S2:** Read depth profiles of contigs flagged as contaminants by the FCS. Colored lines show coverage for each flagged contigs.
**Figure S3:** Locus level read depth density plots.
**Figure S4:** The MCC tree. Maximum clade credibility (MCC) tree from BEAST2 based on the concatenated mitochondrial coding sequences of six taxa.
**Table S1:** Summary of Foreign Contamination Screen (FCS‐GX) results for the *C. formosanus* assembly. The table lists putatively exogenous sequences identified by the NCBI FCS‐GX pipeline.

## Data Availability

Raw sequence reads are deposited in the SRA (BioProject PRJNA1379464) (https://www.ncbi.nlm.nih.gov/bioproject/?term=PRJNA1379464).
