# Supplementary material for: Chromosome‐Level Reference Genome of an Endemic, Endangered Long‐Armed Scarab (Cheirotonus formosanus): Discovery of a Putative Y‐Linked Scaffold and Demographic History
Source: Ecol Evol. 2026 Apr 14;16(4):e73483. doi: 10.1002/ece3.73483 (PMC13077445; doi:10.1002/ece3.73483)
Supplement: Supplementary file 1 — Figure S1: GenomeScope k‐mer profile from PacBio HiFi reads (k = 21). This shows the k‐mer distribution and inferred estimated genome size. Figure S2: Read depth profiles of contigs flagged as contaminants by the FCS. Colored lines show coverage for each flagged contigs. Figure S3: Locus level read depth density plots. Figure S4: The MCC tree. Maximum clade credibility (MCC) tree from BEAST2 based on the concatenated mitochondrial coding sequences of six taxa. Table S1: Summary of Foreign Contamination Screen (FCS‐GX) results for the C. formosanus assembly. The table lists putatively exogenous sequences identified by the NCBI FCS‐GX pipeline. [file ECE3-16-e73483-s001.docx]

### **Supplementary**

| #seq_id | start_pos | end_pos | seq_len | action | div | agg_cont_cov | p_tax_nameto |
| --- | --- | --- | --- | --- | --- | --- | --- |

| ptg000013l | 1 | 67137 | 67137 | EXCLUDE | fung:budding yeasts | 66 | *Starmerella apicola* |
| --- | --- | --- | --- | --- | --- | --- | --- |
| ptg000015l | 1 | 61750 | 61750 | EXCLUDE | fung:budding yeasts | 64 | *Starmerella apicola* |
| ptg000028l | 1 | 21150 | 21150 | EXCLUDE | fung:budding yeasts | 84 | *Starmerella ratchasimensis* |
| ptg000029l | 1 | 55829 | 55829 | EXCLUDE | ​​fung:budding yeasts | 78 | *Starmerella kuoi* |
| ptg000030l | 1 | 33528 | 33528 | EXCLUDE | fung:budding yeasts | 55 | *Starmerella riodocensis* |
| ptg000034l | 1 | 46186 | 46186 | EXCLUDE | fung:budding yeasts | 68 | *Starmerella apicola* |
| ptg000042l | 1 | 35827 | 35827 | EXCLUDE | fung:budding yeasts | 66 | *Starmerella apicola* |
| ptg000046l | 1 | 32902 | 32902 | EXCLUDE | fung:budding yeasts | 59 | *Starmerella apicola* |
| ptg000047l | 1 | 28405 | 28405 | REVIEW | fung:budding yeasts | 20 | *Starmerella riodocensis* |
| ptg000049l | 1 | 36427 | 36427 | EXCLUDE | fung:budding yeasts | 70 | *Starmerella magnoliae* |

Table S1. Summary of Foreign Contamination Screen (FCS-GX) results for the *C. formosanus* assembly. The table lists putatively exogenous sequences identified by the NCBI FCS-GX pipeline.

###
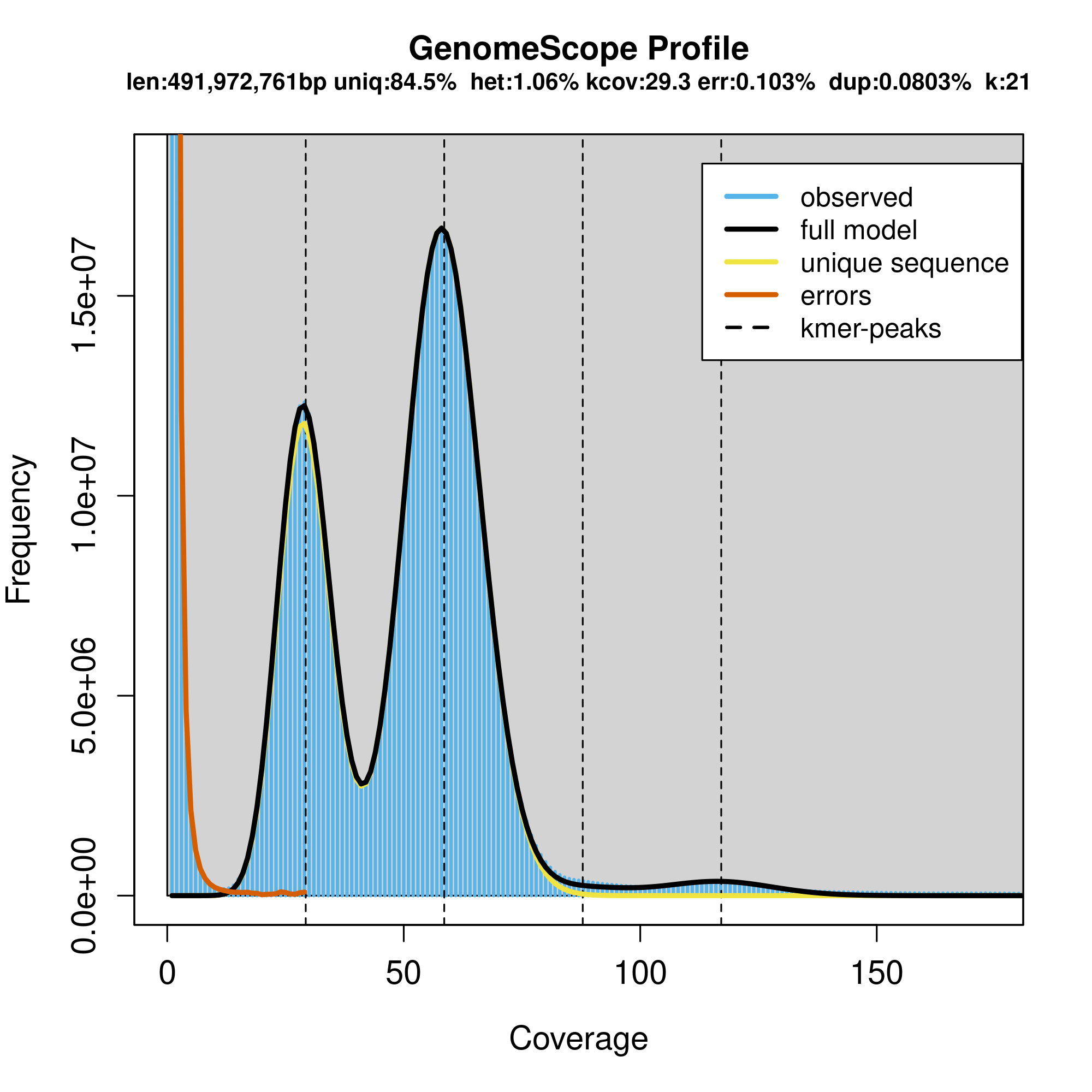

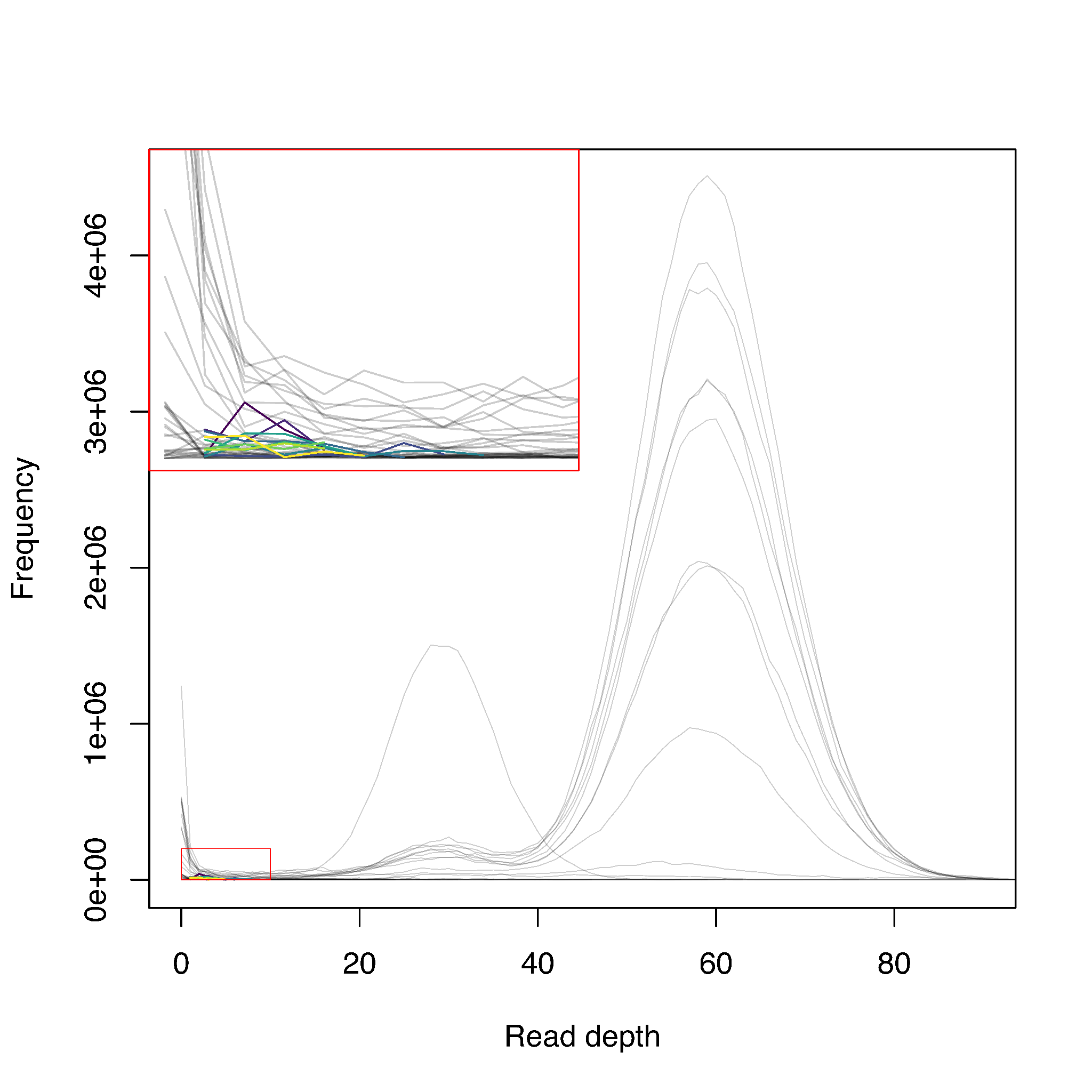


Figure S1. GenomeScope k-mer profile from PacBio HiFi reads (k = 21). This shows the k-mer distribution and inferred estimated genome size.

Figure S2. Read depth profiles of contigs flagged as contaminants by the FCS. Colored lines show coverage for each flagged contigs. The most of contigs has read depth near 58x, with subset near 29x (sex specific). All contaminated contigs were removed prior to scaffolding.

###
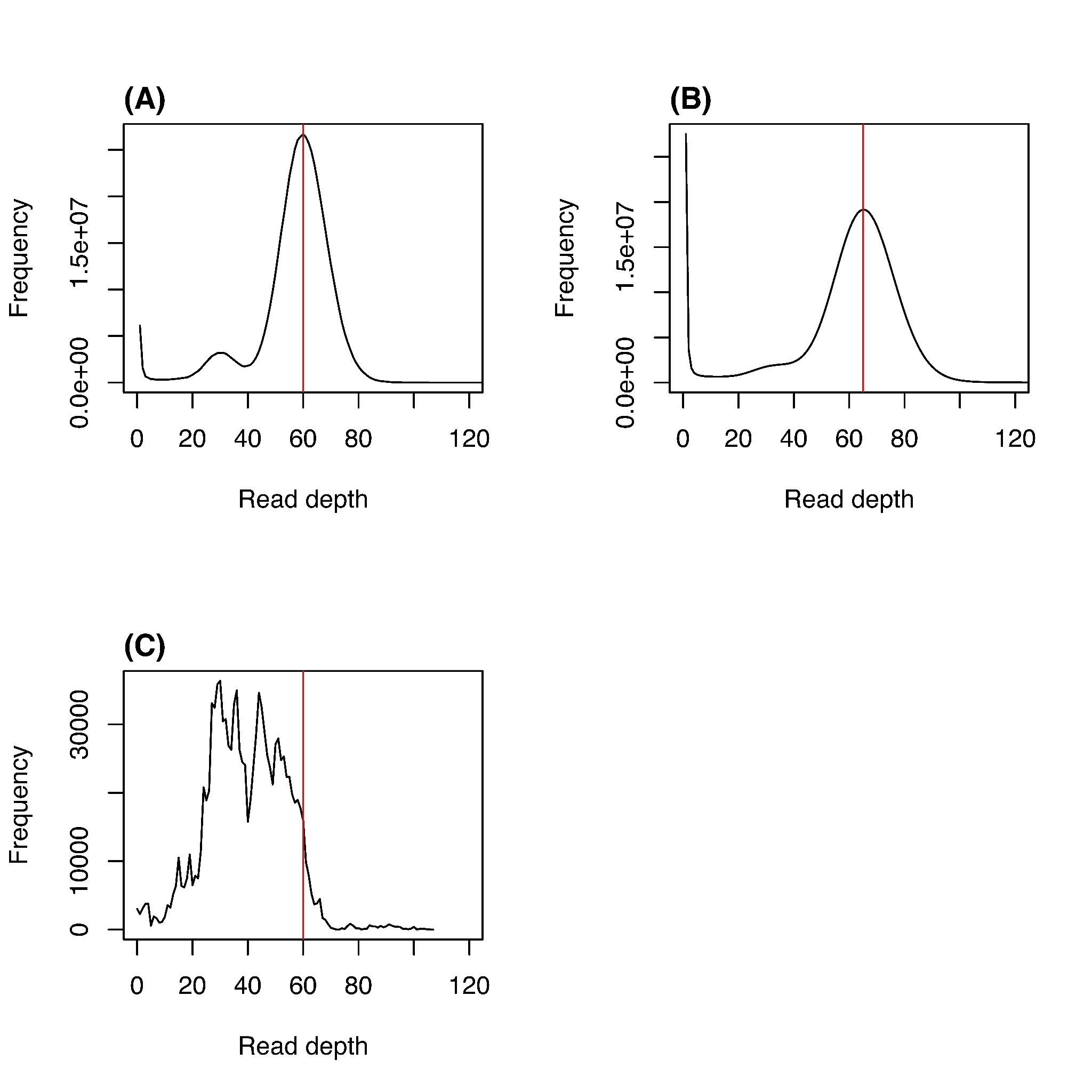

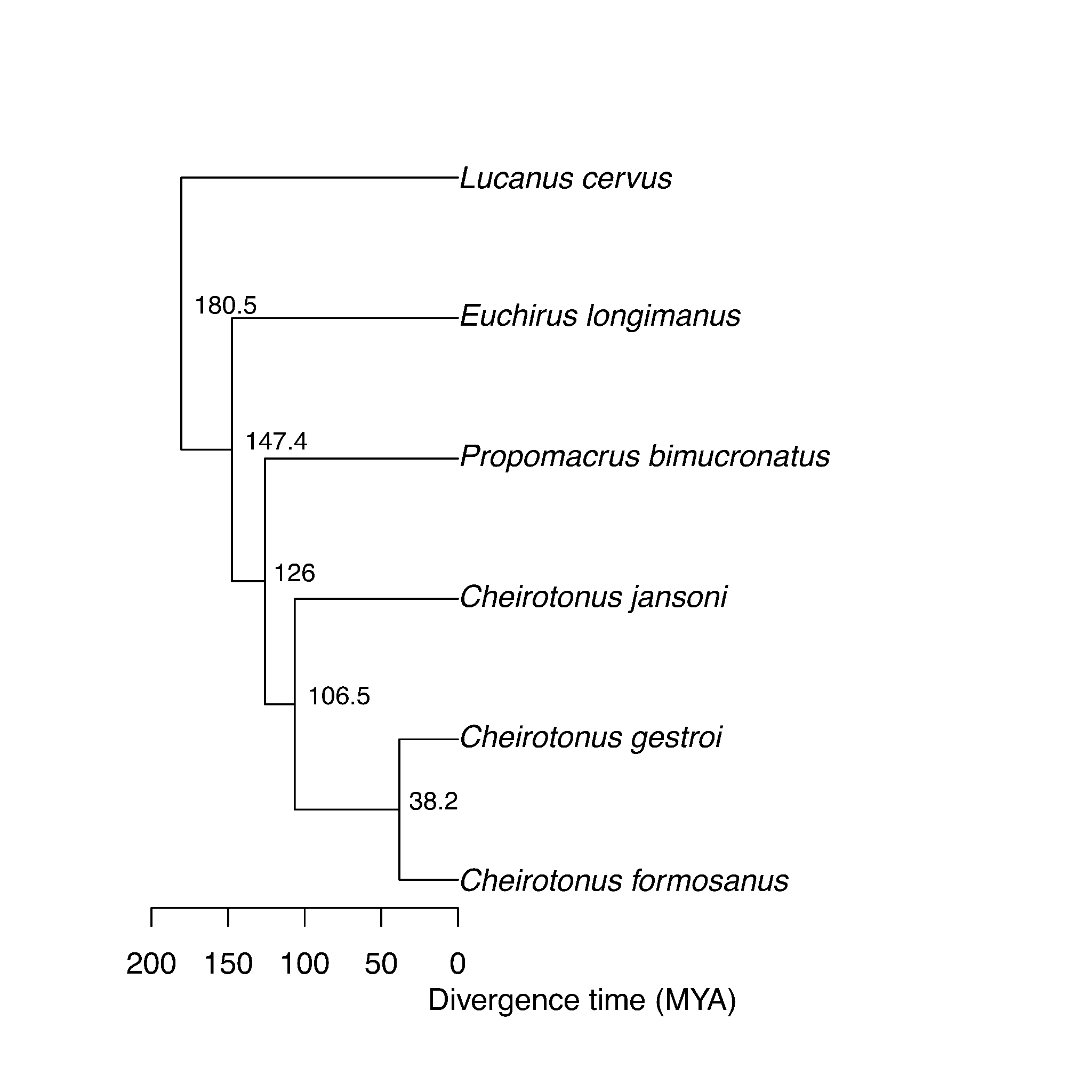


Figure S3. Locus level read depth density plots. A) Male (PacBio HiFi) genome wide coverage. The modal depth is ~60x. B) Female (Illimina PE150) genome wide coverage. The modal depth is~65x. C) Male coverage on scaffold 13 (putative Y-linked). The depth is approximately half the whole genome mode, consistent with the hemizygous single-copy Y sequence.

Figure S4. The MCC tree. Maximum clade credibility (MCC) tree from BEAST2 based on the concatenated mitochondrial coding sequences of six taxa. *C. formosanus* is recovered as sister to *C. gestroi,* rather than C. jansoni. The posterior mean MRCA age for *C. formosanus* and *C. gestroi* was 38.2 MYA (95% HPD: 32.1 - 44.8 MYA). The divergence between *C. jansoni* and *C. formosanus* and *C. gestroi* was 106.47 MYA (95% HPD 89.7 - 123.2 MYA). The unit is time millions of years before present.
